# Evaluation of Proton MR Spectroscopy for the Study of the Tongue Tissue in Healthy Subjects and Patients With Tongue Squamous Cell Carcinoma: Preliminary Findings

**DOI:** 10.3389/froh.2022.912803

**Published:** 2022-07-18

**Authors:** Salem Boussida, Yvener François, Adrien Heintz, Zuzana Saidak, Stéphanie Dakpé, Alexandre Coutte, Bruno Chauffert, Bernard Devauchelle, Antoine Galmiche, Sylvie Testelin, Patrick Goudot, Jean-Marc Constans

**Affiliations:** ^1^Radiology Department, University Hospital of Amiens Picardie, Amiens, France; ^2^CHIMERE UR 7516 Research Team for Head & Neck, Institute Faire Faces, University of Picardie Jules Verne, Amiens, France; ^3^Faculty of Medicine, Assistance Publique - Hôpitaux de Paris (AP-HP), Sorbonne University, Paris, France; ^4^Department of Biochemistry, University Hospital of Amiens, Amiens, France; ^5^Department of Maxillofacial Surgery and Stomatology, University Hospital of Amiens, Amiens, France; ^6^Department of Radiotherapy, University Hospital of Amiens, Amiens, France; ^7^Department of Medical Oncology, University Hospital of Amiens, Amiens, France

**Keywords:** tongue tissue, squamous cell carcinoma, proton magnetic resonance spectroscopy, ^1^H-MRS, spectroscopic and metabolic profile, noninvasive biomarkers

## Abstract

**Purpose:**

To noninvasively assess spectroscopic and metabolic profiles of healthy tongue tissue and in an exploratory objective in nontreated and treated patients with tongue squamous cell carcinoma (SCC).

**Methods:**

Fourteen healthy subjects (HSs), one patient with nontreated tongue SCC (NT-SCC), and two patients with treated tongue SCC (T-SCC) underwent MRI and single-voxel proton magnetic resonance spectroscopy (^1^H-MRS) evaluations (3 and 1.5T). Multi-echo-times ^1^H-MRS was performed at the medial superior part (MSP) and the anterior inferior part (AIP) of the tongue in HS, while ^1^H-MRS voxel was placed at the most aggressive part of the tumor for patients with tongue SCC. ^1^H-MRS data analysis yielded spectroscopic metabolite ratios quantified to total creatine.

**Results:**

In HS, compared to MSP and AIP, ^1^H-MRS spectra revealed higher levels of creatine, a more prominent and well-identified trimethylamine-choline (TMA-Cho) peak. However, larger prominent lipid peaks were better differentiated in the tongue MSP. Compared to HS, patients with NT-SCC exhibited very high levels of lipids and relatively higher values of TMA-Cho peak. Interestingly, patients with T-SCC showed almost nonproliferation activity. However, high lipids levels were measured, although they were relatively lower than lipids levels measured in patients with NT-SCC.

**Conclusion:**

The present study demonstrated the potential use of *in-vivo* ^1^H-MRS to noninvasively assess spectroscopic and metabolic profiles of the healthy tongue tissue in a spatial location-dependent manner. Preliminary results revealed differences between HS and patients with tongue NT-SCC as well as tongue T-SCC, which should be confirmed with more patients. ^1^H-MRS could be included, in the future, in the arsenal of tools for treatment response evaluation and noninvasive monitoring of patients with tongue SCC.

## Introduction

Magnetic resonance spectroscopy (MRS) is a noninvasive quantitative tool that is based on a spectroscopic analysis of tissue metabolism. MRS can provide molecular analysis of biochemical composition and evaluate the presence of specific metabolites in the tissue being investigated. Proton MRS (^1^H-MRS), the most used MRS method in clinical practice, has mostly been applied to study brain pathologies, particularly brain tumors for diagnosis, differentiation between nonmalignant and malignant tissues, prognosis, and evaluation of treatment responses [1].

Thanks to technological advances in both the software and hardware, clinical applications of ^1^H-MRS have been applied to other organs, than the brain, including for diagnosis of cancer in the breast [2], prostate [3], liver [4], and, more recently, musculoskeletal system [5]. The value of ^1^H-MRS in skeletal muscle was clearly established [6], providing information on the metabolic properties (energy, lipids) of muscles. These parameters have the potential to be used as biomarkers to detect pathological processes and to monitor, for example, patients with head and neck (HN) cancer undergoing therapy.

Head and neck squamous cell carcinoma (SCC) is the sixth most common cancer type in the world with 890,000 new cases and 450,000 deaths in 2018. The incidence of SCC is expected to increase by 30% (being 1.08 million new cases per year) by 2030 [Global Cancer Observatory (GLOBOCAN)] and is especially frequent in the northeast France [7, 8]. SCC can occur in various sublocations of the upper aerodigestive tract, including the pharynx, larynx, sinuses, nasal cavity, and the oral cavity with the most common subsite being the oral tongue, which is also one of the worst subsites in terms of prognosis [9]. SCC could be a very aggressive cancer and has a bad prognosis if not detected early and, thus, is associated with high mortality. The development of simple and reliable biomarkers for the early detection of SCC is one of the solutions to better diagnose, treat these tumors, evaluate and monitor treatment combinations, and, hence, reduce mortality.

In this context, ^1^H-MRS, being a noninvasive, rapid, informative, and quantitative technique, with a demonstrated higher sensitivity compared to MRI [1], has great potential to help with early cancer detection, diagnosis, and treatment response evaluation.

Only a few previous ^1^H-MRS studies (both *in-vivo* [10, 11] and *ex-vivo* [12]) have been performed with a limited number of subjects. Moreover, to the best of our knowledge, no previous study has focused on the metabolic profiles of the tongue region in healthy subjects. This could be related to some difficulties specific to the HN region, as well as to the lack (in the past) of adequate methods of spectroscopic data acquisition. Indeed, performing *in-vivo* MRS studies on HN sites is challenging. Magnetic susceptibility artifacts arising from tissue–bone–air interfaces, as well as motion artifacts related to respiration and swallowing are the main limitations of performing *in-vivo* MRS studies on the HN region. However, the tongue region, one of the most common sublocations of SCC (95% of tongue tumors), is mainly constituted of muscles and is a relatively homogeneous structure in healthy subjects and could be suitable for *in-vivo* spectroscopic measurements.

Thus, the main objective of this study was to noninvasively assess spectroscopic and metabolic profiles of the healthy tongue tissue in two different spatial locations (the medial superior and the anterior inferior parts of the tongue) in healthy subjects. As a second exploratory objective, we challenged the potential use of ^1^H-MRS in differentiating normal tongue tissue from SCC before and after treatment.

## Methods

### Subjects

Fourteen healthy subjects (HSs) were recruited from the Institut “Faire Faces” (Amiens, France). Three patients with SCC were recruited from the Oral and Maxillofacial Surgery department (Amiens University Hospital). One patient with nontreated tongue SCC (NT-SCC) (TNM stage: pT4aN0MxR0); one patient with treated tongue SCC [T-SCC; TNM stage: P16-, T4 N2b Mx, chemotherapy: carboplatin and 5-fluorouracil (5FU) delivered on 3 days, radiotherapy: a curative dose of 69.96 Gy delivered on 33 fractions for the tumor, and a prophylactic dose of 54.12 Gy delivered on 33 fractions]; and patient with T-SCC [TNM stage: T3 N0 M0, chemotherapy: cisplatin (CDDP) 100 mg/m^2^ delivered on 3 days, and radiotherapy: a dose of 70 Gy delivered on 35 fractions for the tumor]. The staging was based on the 8th edition of the TNM staging system of the Union for International Cancer Control (UICC). Ethical approval for this study was obtained from Clermont-Ferrand Ethical Committee (2018-A02389-46) and written informed consent was obtained from all the subjects before the study.

### Data Acquisition

Patients with HS and SCC underwent MRI and proton magnetic resonance spectroscopy (^1^H-MRS) using, respectively, Achieva dStream 3T (Philips Healthcare, Best, Netherlands) and GE Optima MR450w 1.5T (GE Healthcare, USA) MRI scanners, using, respectively, a 32- and 24-channel head and neck coil.

**Figure 1:**
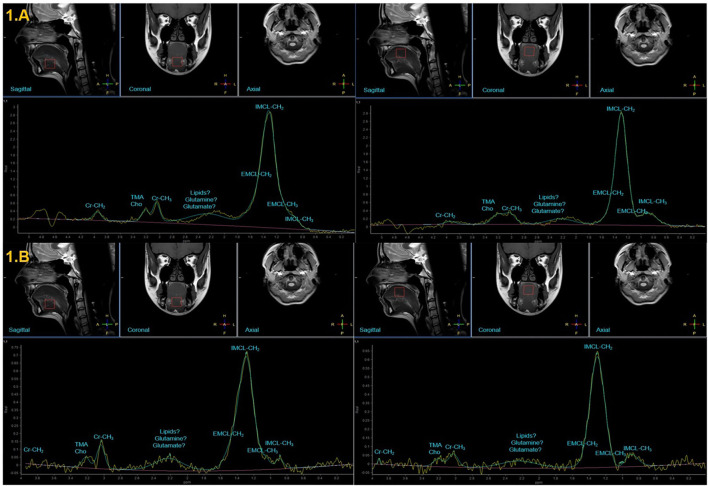
Representative MR images illustrating ^1^H-MRS voxel placement at the medial superior and the anterior inferior parts of the tongue in healthy subjects and their corresponding representative MR spectra measured at TE of 35 ms **(A)** and TE of 144 ms **(B)**.

Patients with HS and SCC underwent the same MRI acquisitions [three-dimensional (3D) T1-weighted spin-echo without contrast enhancement, gradient-echo T2^*^, T2-weighted imaging with fat saturation, and diffusion-weighted imaging (DWI)] except that patients with SCC had, in addition, a 3D T1-weighted spin-echo with contrast enhancement. ^1^H-MRS acquisitions consisted of a single-voxel Point RESolved Spectroscopy (PRESS) sequence with two echo times (TE = 35 and 144 ms) performed using a volume voxel of 2 cm^3^ × 2 cm^3^ × 2 cm^3^. ^1^H-MRS voxel was placed at two different locations: the medial superior part (MSP) and the anterior inferior part (AIP) of the tongue for healthy subjects (Figure 1), while it was placed at the most aggressive part of the tumor (based on the appearance of the lesions in pre- and postcontrast-enhanced 3D T1 MRI and T2-weighted imaging with fat saturation).

### Data Analysis

Proton magnetic resonance spectroscopy data from HS were analyzed using the MR SpectroView Analysis package from Philips Achieva dStream 3.0T TX. ^1^H-MRS data from patients with T-SCC and NT-SCC were analyzed on the SUN imaging workstation (Advantage Windows) using SAGE (Spectroscopic Analysis, GE). Before analysis, the quality of ^1^H-MRS spectra was assessed (signal-to-noise ratio, spectral resolution, and estimation of full width at half maximum on water peak). ^1^H-MRS spectra obtained from the AIP of the tongue were all (14) of good quality. However, only six of 14 ^1^H-MRS spectra that were obtained from the MSP of the tongue fitted quality criteria and were included in the analysis step.

Data analysis was performed in the time domain directly on free induction decays (FIDs). Because the tongue is mainly composed of interlacing skeletal muscle and pockets of adipose tissue, the spectroscopic profile of the healthy tongue was expected to be close to the well-studied skeletal muscle spectroscopic profile [6, 13]. Hence, for metabolite quantification, we selected the most relevant resonances measurable in skeletal muscle. These resonances were specified and described by Gaussian line shapes in the frequency domain and represented as follows: intramyocellular lipid-methyl (IMCL-CH_3_) resonance at 0.9 ppm, extramyocellular lipid-CH_3_ (EMCL-CH_3_) resonance at 1.1 ppm, intramyocellular lipid-methylene (IMCL-CH_2_) resonance at 1.3 ppm, extramyocellular lipid-CH_2_ (EMCL-CH_2_) resonance at 1.45–1.5 ppm, creatine-CH_3_ (Cr-CH_3_) resonance at 3.03 ppm, Cr-CH_2_ resonance at 3.92 ppm, trimethylamine (TMA) and choline (Cho) resonances at 3.20–3.22 ppm, and the water resonance being centered at 4.72 ppm.

Spectroscopic metabolites were then quantified as ratios to total creatine (tCr) with tCr representing the sum of Cr-CH_2_ and Cr-CH_3_. Mean values (±SD) of metabolite ratios were calculated for HS (the MSP and the AIP parts of the tongue) and patients with NT-SCC and T-SCC.

## Results

The present study demonstrated the potential use of the ^1^H-MRS technique to noninvasively quantify the spectroscopic and metabolic profiles of the tongue tissue in HS. Preliminary findings from patients with NT-SCC and T-SCC are also presented.

**Figure 2:**
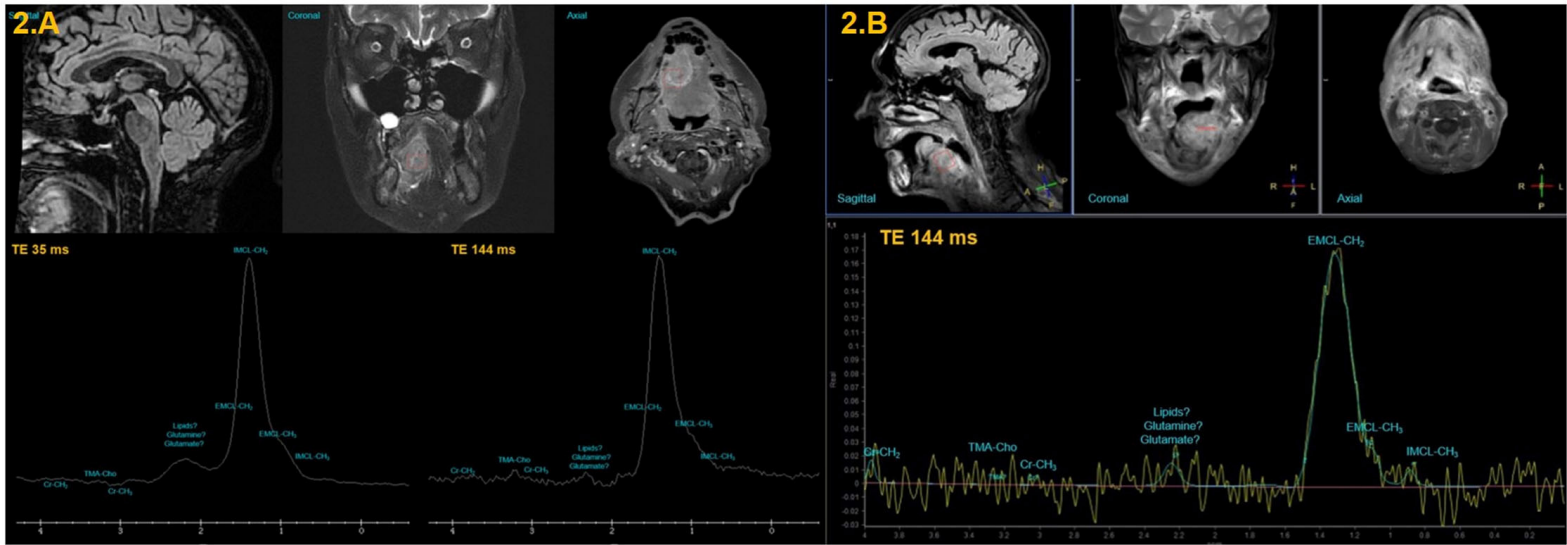
MR images illustrating ^1^H-MRS voxel placement in the nontreated tongue SCC **(A)** and treated tongue SCC **(B)** and their representative ^1^H-MRS spectra.

Representative MR images illustrating ^1^H-MRS voxel placement at the MSP and the AIP of the tongue in HS are shown on the top of Figures 1, 2. Two corresponding representatives MR spectra obtained from the MSP and the AIP of the tongue in HS are shown at the bottom of Figures 1, 2.

The spectrum obtained from the MSP of the tongue in HS (Figure 1A) showed a very prominent and large peak (from 1.1 to 1.5 ppm) centered at 1.3 ppm arising from protons in the methylene (CH_2_) groups of lipids that are attributed to IMCL-CH_2_. A less prominent peak, resulting from protons in the methyl (CH_3_) groups of lipids corresponding to IMCL-CH_3_, is observed at 0.9 ppm. A weak and relatively large nonassigned peak centered at 2.3 ppm is observed. Cr-CH_3_ peak is observed at 3.03 ppm. TMA-Cho peak is measured at 3.21 ppm and Cr-CH_2_ peak is measured at 3.92 ppm.

The spectrum obtained from the AIP of the tongue in HS (Figure 1A) also showed a very prominent and large peak (from 1.1 to 1.5 ppm) centered at 1.3 ppm arising from IMCL-CH_2_ that should attributed to IMCL-CH_3_ and centered at 0.9 ppm. A weak but larger (compared to the MSP of the tongue) nonassigned peak centered at 2.3 ppm is observed. A higher Cr-CH_3_ peak is also observed at 3.03 ppm. A more prominent and well-separated TMA-Cho peak is observed at 3.21 ppm and, finally, a higher Cr-CH_2_ peak is observed at 3.92 ppm.

In patient with NT-SCC, the spectrum obtained from voxel placed at the most aggressive part of the SCC of the tongue (Figure 2A) showed a very prominent peak with a larger extent (compared to HS; from 1 to 1.8 ppm) centered at 1.3 ppm arising from IMCL-CH_2_ with a shoulder attributed to IMCL-CH_3_ and centered at 0.95 ppm. A weak and relatively large nonassigned peak centered at 2.3 ppm is observed. A reduced Cr-CH_3_ peak is observed at 3.03 ppm. However, a higher TMA-Cho peak is measured at 3.21 ppm that is more visible at TE of 144 ms (Figure 2A) and a weaker Cr-CH_2_ peak is measured at 3.92 ppm.

In patients with T-SCC, spectra obtained from the MSP of the tongue (Figure 2B) showed a very prominent with smaller extent compared to patient with NT-SCC (from 1.1 to 1.5 ppm) centered at 1.3 ppm arising from IMCL-CH_2_. Spectra also contained a separate and less prominent peak, resulting from IMCL-CH_3_, observed at 0.9 ppm. A weak and relatively large nonassigned peak centered at 2.3 ppm is observed. A weaker Cr-CH_3_ peak is observed at 3.03 ppm. Furthermore, a very low-intensity TMA-Cho peak is measured at 3.21 ppm and Cr-CH_2_ peak is measured at 3.92 ppm.

## Discussion

The main objective of this study was to noninvasively assess spectroscopic and metabolic profiles of the tongue tissue in two different spatial locations (the MSP and the AIP of the tongue) in healthy subjects (HSs). As a second exploratory objective, we challenged the potential use of ^1^H-MRS in differentiating normal tongue tissue from tongue SCC before and after treatment.

**Figure 3:**
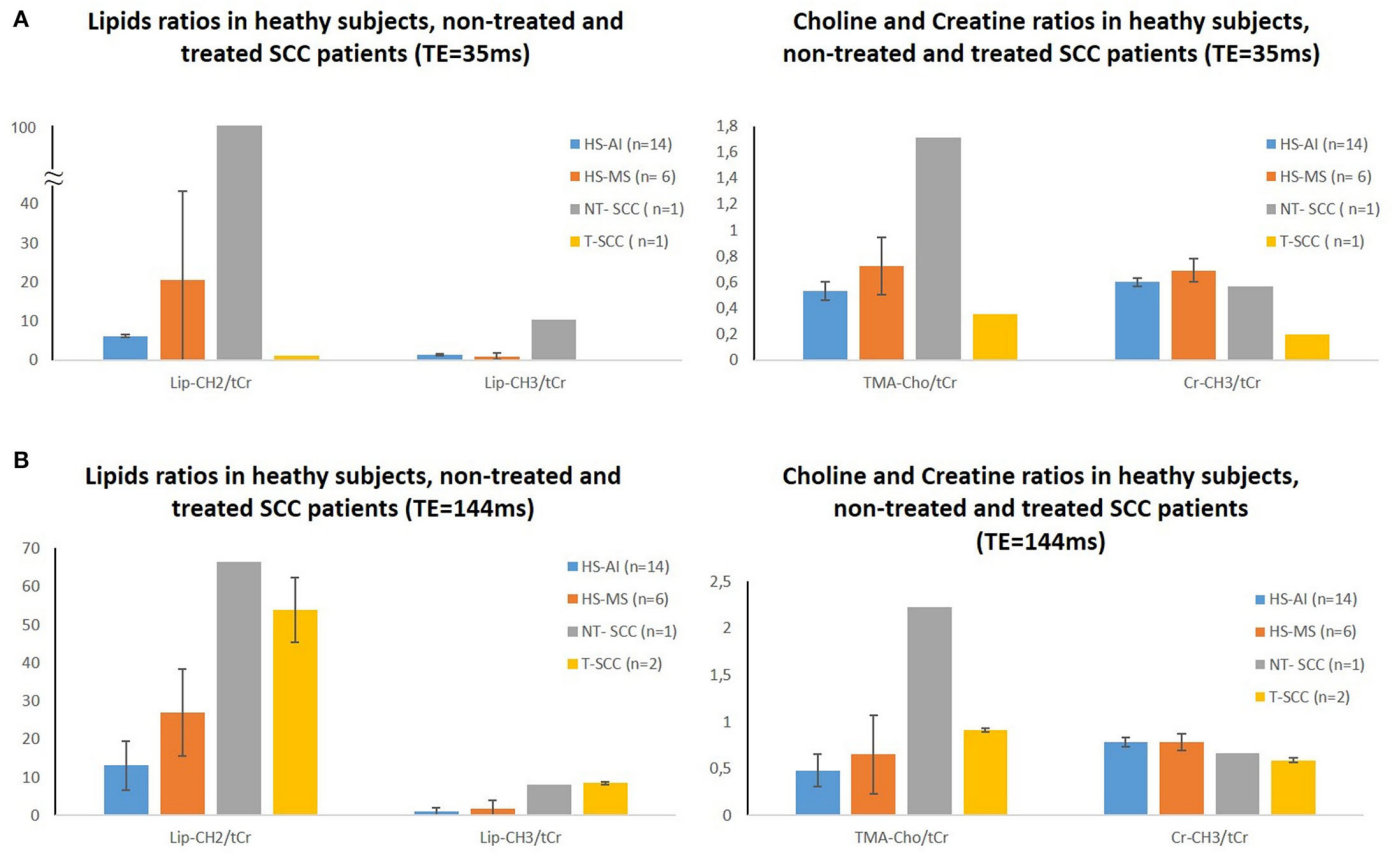
Lipids, choline, and creatine metabolites ratios differences between healthy subjects and patients with nontreated and treated SCC measured at TE = 35 ms **(A)** and TE = 144 ms **(B)**. Note that Lip-CH_2_ represents IMCL-CH_2_ and IMCL-CH_2_ and Lip-CH_3_ represents IMCL-CH_3_ and IMCL-CH_3_. HS-AI, healthy subjects-anterior inferior part of the tongue; HS-MS, healthy subjects-medial superior part of the tongue; NT-SCC, patient with nontreated SCC; T-SCC, patient with treated SCC.

The present study demonstrated the ability of the ^1^H-MRS technique to noninvasively quantify the spectroscopic and metabolic profiles of the tongue tissue in HS and highlighted differences in spectroscopic profiles between HS and patients with nontreated tongue SCC as well as treated tongue SCC (Figure 3).

Because the type and compositions of the muscles of the MSP and AIP (genioglossus muscles) parts of the tongue are different and because SCC lesions most often arise at the borders of the tongue before affecting the body [14], one can expect metabolic differences between the MSP and the AIP parts of the tongue. For this reason, the first objective of this study was to assess *in-vivo* single-voxel ^1^H-MRS measurements, in HS, at two different locations, i.e., the MSP and the AIP of the tongue. As described in the Results section, differences were observed in HS between the spectroscopic profiles of these two locations.

One important difference concerned lipid levels (Figure 3). In previous *in-vivo* ^1^H-MRS studies of skeletal muscle [15], lipid peaks were well identified and separated with more subpeaks arising separately from EMCL and IMCL protons. In our study, it was not possible to separate IMCL and EMCL from both CH_2_ and CH_3_ in a reproducible way. This could be explained by different reasons, mainly tongue motion (especially at the MSP), magnetic susceptibility effects, and residual dipolar coupling that can affect metabolites peaks separation [13]. Moreover, differences in terms of muscle composition between the tongue and skeletal muscles, as well as muscle fiber orientations can also affect spectral resolution. This is consistent with previous studies [16] reporting that peaks of EMCL and IMCL in calf muscles are separated when fibers are orthogonally oriented to the main magnetic field (B0), while nonseparated peaks are observed when muscles are oriented parallel to B0.

Further differences between the MSP and the AIP of the tongue were also observed in levels of Cr peaks (Figure 3), an important metabolite that is often used as a reference to estimate relative spectroscopic concentrations of all other metabolites. The AIP of the tongue showed higher levels in both the Cr-CH_3_ (at 3.03 ppm) and Cr-CH_2_ (at 3.92 ppm) compared to the MSP of the tongue. Cr reflects the energy metabolism and the cellularity index of the tissue; this difference may be attributed to the higher level of muscle composition in the AIP (the genioglossus muscles) compared to the MSP (with less muscle volume) of the tongue and their different functions: the anterior inferior muscles are more implicated in motor function, while the superior muscles are rather implicated in sensitive, gustative, and food transport (providing a smooth surface for food to slide into the hypopharynx) functions in the MSP of the tongue.

Finally, we noticed a better separation between the TMA-Cho peak and the Cr-CH_3_ peak in the AIP compared to the MSP of the tongue. This point is very important since it offers the ability to better measure and follow-up the TMA-Cho peak (particularly the Cho/Cr ratio, a cell proliferation marker) in tumor conditions.

In summary, the spectroscopic and metabolic profiles of the AIP of the tongue seem to be closer to skeletal muscle profiles compared to the MSP of the tongue. This is likely in agreement with the higher level of muscle composition in the AIP of the tongue and its main function, i.e., motor function. The presented differences, observed in HS, are important and should be taken into account in the spectroscopic evaluation and follow-up of SCC lesions.

The second exploratory objective of this study was to challenge the potential of *in-vivo* ^1^H-MRS in differentiating healthy tongue tissue from SCC before and after treatment. The preliminary presented results suggest important differences in the spectroscopic profiles between HS and patients with NT-SCC and T-SCC. In NT-SCC (Figure 2A), the TMA-Cho/tCr ratio showed a higher value compared to HS. Although protons related to the Cho peak overlap with protons related to the TMA peak, the increase in the complex TMA-Cho is probably associated with an increase in Cho-containing metabolites peak [mainly phosphatidylcholine (PC) and glycerophosphocholine (GPC)] rather than TMA peak. Indeed, Cho (and its derivatives) is an important constituent in phospholipid metabolism of the cell membranes and is identified as a cell tumor proliferation marker. In clinical practice, Cho/Cr is the most studied ratio for the prognosis of brain tumors [17]. Elevated Cho/Cr is also in agreement with a previous *in-vitro* ^1^H-MRS study [18], which showed that Cho/Cr ratio level was significantly higher in the SCC tongue than in normal tissue or posttherapeutic tissue. Moreover, in our study, patient with NT-SCC showed a Cho/Cr ratio of 2. This ratio is relatively low compared to Cho/Cr ratio levels that we can usually measure in brain tumors, such as in glioblastomas (Cho/Cr = from 3 to 6), in meningiomas (Cho/Cr = from 3 to 10, with a concomitant decrease in Cr levels), and medulloblastomas (Cho/Cr = from 3 to 16) [1]. We hypothesize that such a difference could be related to the epidermoid type of SCC tumor itself, which may have lower mitotic activity and cell membrane turnover compared to tumors located in the conjunctive and supporting tissues such as in the glial (glioblastomas), meningeal (meningiomas), and medulloblastomas cells. Interestingly, patients with T-SCC showed almost no proliferation activity (with Cho/Cr ratio <1.5) after radiotherapy or radiochemotherapy treatments. This could be explained by either the efficiency of the treatment and/or the relatively low level of proliferation before treatment (Cho/Cr = 2) or by the intersubject treatment (chemotherapy) variability.

Another interesting finding concerned the variation of lipid levels. Broadening of IMCL-CH_2_ (centered at 1.3 ppm) and IMCL-CH_3_ (centered at 0.95 ppm) peaks combined with their increased FWHM values tend to indicate an increased amount of phospholipids. In patient with NT-SCC, this could be related to increased necrotic lipids as a result of an inadequate balance between vascularization and cell proliferation and may be responsible for metabolic stresses such as hypoxia and energy deprivation as in solid tumors [1]. However, ^1^H-MRS measurements in patient with NT-SCC showed a relatively low level of proliferation (Cho/Cr ratio of 2) and could not be the only explanation for such high lipid levels. Thus, more patients with SCC and further complementary measurements are needed to investigate the tumor vascularization environment using quantitative methods, such as dynamic contrast-enhanced MRI (DCE-MRI) to take benefit from its derived pharmacokinetic parameters [19].

In contrast, ^1^H-MRS measurements in patients with T-SCC showed a lower level of necrotic lipids compared to patients with NT-SCC (Figure 2B). This result was unexpected and is not in agreement with what we can usually measure in brain tumors, especially in glioblastomas where very high levels of necrotic lipids are usually detected [1]. We are unable to explain such results, since many variable factors could interfere (intersubject treatment variability). This emphasizes the interest of further metabolic studies with different time point measurements, particularly before and after each treatment step, in order to separate treatment effects from all other sources of variability. Further, a decrease in the level of the creatine peak was observed in patients with T-SCC compared to HS. One explanation would be an increased metabolic rate, thus increased energy and energetic reserves consumption of creatine and phosphocreatine. This phenomenon is known to be particularly observed under treatments or in tumors that are with high proliferation rate [1].

Another finding in the present study was the probable presence of NMR visible polyamines and/or amino acid resonances [between Cr-CH_3_ (3.03) and TMA-Cho (3.21 ppm)] peaks in NT-SCC. The amino acid-derived polyamines have long been associated with cell growth and cancer and specific oncogenes and tumor suppressor genes regulate polyamine metabolism. Upregulation of polyamine has been found to increase cell proliferation, decrease apoptosis, and promote tumor invasion and metastasis [20]. Such resonances, indicating changes in polyamines that are usually observed in prostate cancer [21], need to be better studied and may be used to help to monitor treatment responses in patients with SCC.

Finally, we noticed in all the ^1^H-MRS spectra (HS, NT-SCC, and T-SCC subjects), the presence of nonassigned resonances between 2 and 2.4 ppm that could be attributed to either lipid or glutamine-glutamate resonances [22] increase. Further studies using 2-dimensional total correlated proton nuclear magnetic resonance spectroscopy (TOCSY) could be of great interest. TOCSY is a technique that was demonstrated to be able to identify and quantify a wide range of metabolites such as amino acids, peptides, triglycerides, and phospholipids precursors [23]. Moreover, high-resolution magic angle spinning (HRMAS) NMR spectroscopy techniques [24] on tongue SCC tumor biopsies may also bring more insights into these resonance changes, especially for glutamine metabolite, that are usually related to malignancy or tumor evolution.

The present study has some limitations: (1) Because of the differences in both the muscle content and muscle fibers orientation and motion artifacts (respiration and swallowing), *in-vivo* ^1^H-MRS spectra obtained from the tongue MSP were not of sufficiently high quality and only six (out of 14) HS were retained and used for metabolites ratios quantifications in the tongue MSP, while all the fourteen ^1^H-MRS spectra obtained from the tongue AIP were included; (2) SCC tumors (especially T-SCC) showed higher tissue heterogeneity, compared to healthy tongue tissue, reducing, consequently, the quality of ^1^H-MRS spectra; and (3) Although the present study was mainly focused on studying healthy tongue tissue, the small number of patients with SCC was a major limitation. We only recruited three patients with tongue SCC to preliminary investigate the potential use of ^1^H-MRS in differentiating normal tongue tissue from SCC before and after treatment. The small SCC patients' sample did not allow any generalization, but we hope that our preliminary results could serve as a starting point for future studies with larger samples and screening patients with SCC before and after each treatment step to validate the present results. Combining ^1^H-MRS data with other relevant imaging tracers demonstrated in studies dealing with brain tumors, such as ^11^C-methionine [25] and 3′-deoxy-3′-(18)F-fluorothymidine ((18)F-FLT) [26] (cell proliferation metabolism), and [F-18] Fluoromisonidazole (tumor hypoxia) [27] using PET imaging, will be of a high added value.

Despite these limitations, the present study opens up new perspectives in the study of SCC lesions. ^1^H-MRS studies could be compared and/or coupled to quantitative imaging techniques, such as [F18]-fluoro-2-deoxy-D-glucose-PET (18F-FDG-PET). Together, ^1^H-MRS and 18F-FDG-PET have a very high sensitivity compared to MRI and X-ray CT [28] and may be very helpful in determining the extension, depth of invasion of the tumor, and prognosis evaluation in patients with oral SCC [29].

Furthermore, in line with the objective of the present study, *in-vivo* ^1^H-MRS metabolic signatures could be coupled to *in-vitro* ^1^H-MRS analysis studies of accessible biofluids such as serum and saliva. Previous metabolomics studies revealed the existence of metabolic signatures in such biofluids with several tumor-specific metabolites that could discriminate oral cancer from healthy controls or even precancerous lesions [30]. In this objective, serum, an easily accessible biofluid from a blood sample, could be concomitantly analyzed (without preparation [30]) with ^1^H-MRS acquisitions. Previous blood samples from oral cancer patients were analyzed and exhibited altered metabolic profiles occurring at an early stage of cancer [31], characterized mainly by an altered energy metabolism with the presence of ketone bodies, the suppression of tricarboxylic acid cycle, and abnormal amino acid catabolism. Another previous study [32] on esophageal SCC revealed that plasma phospholipid metabolism plays a critical role in oncogenesis; this result is in agreement with our *in-vivo* results as depicted by a significant increase of lipids in patients with tongue SCC.

Moreover, in a previous NMR spectroscopy study [33], specific variations in the salivary metabolomic profile were identified and revealed that fucose, glycine, methanol, and proline were highly discriminant between patients with HN cancer and control subjects. Further, metabolomics studies revealed high sensitivity to saliva metabolic changes [34] and to DNA methylation alterations (that are detectable in saliva) of patients with oral squamous cell carcinoma (OSCC) [35–37], suggesting that salivary biomarkers are valuable for OSCC early diagnosis and OSCC stratification. Altogether, these elements emphasize the importance of conducting further metabolomics studies with concomitant *in-vivo* and *ex-vivo* biofluids (serum and/or saliva) ^1^H-MRS analyses.

## Conclusion

The present study demonstrated the potential use of the *in-vivo* ^1^H-MRS technique to noninvasively assess the spectroscopic and metabolic profiles of the tongue tissue, in a spatial location-dependent manner, in healthy subjects. Relevant differences, observed in healthy subjects, were presented and should be taken into account in the spectroscopic and metabolic evaluations of SCC lesions. Preliminary results revealed differences between healthy subjects and nontreated as well as treated patients with tongue SCC, which should be confirmed with a higher number of patients. These preliminary findings highlight the potential of ^1^H-MRS to be, in the future, included in the existing arsenal of tools of diagnosis, treatment response evaluation, estimation of the potential of tumor recurrence and patient survival, and, thus, could improve the surgical management of patients with tongue SCC.

## Data Availability Statement

The raw data supporting the conclusions of this article will be made available by the authors, without undue reservation.

## Ethics Statement

Ethical approval for this study was obtained from Clermont-Ferrand Ethical Committee. The patients/participants provided their written informed consent to participate in this study.

## Author Contributions

Conceived and designed the study: SB, J-MC, PG, BD, AC, BC, AG, ST, SD, and ZS. Performed the acquisitions: J-MC. Analyzed the data: SB, J-MC, and AH. Recruitment and management of patients: J-MC, PG, BD, AC, BC, ST, SD, and YF. Wrote the paper: SB and J-MC. All authors contributed to the article and approved the submitted version.

## Funding

The present work was financially supported by the Gueules Cassées Foundation (https://www.gueules-cassees.asso.fr) and the Stomatology and Maxillo-Facial Surgery Service of the Pitié-Salpêtrière hospital.

## Conflict of Interest

The authors declare that the research was conducted in the absence of any commercial or financial relationships that could be construed as a potential conflict of interest.

## Publisher's Note

All claims expressed in this article are solely those of the authors and do not necessarily represent those of their affiliated organizations, or those of the publisher, the editors and the reviewers. Any product that may be evaluated in this article, or claim that may be made by its manufacturer, is not guaranteed or endorsed by the publisher.
